# A Federated Reinforcement Learning Framework via a Committee Mechanism for Resource Management in 5G Networks

**DOI:** 10.3390/s24217031

**Published:** 2024-10-31

**Authors:** Jaewon Jeong, Joohyung Lee

**Affiliations:** School of Computing, Gachon University, Seongnam 13120, Republic of Korea; deviljoe996@gachon.ac.kr

**Keywords:** federated reinforcement learning, virtual instance scaling, committee mechanism

## Abstract

This paper proposes a novel decentralized federated reinforcement learning (DFRL) framework that integrates deep reinforcement learning (DRL) with decentralized federated learning (DFL). The DFRL framework boosts efficient virtual instance scaling in Mobile Edge Computing (MEC) environments for 5G core network automation. It enables multiple MECs to collaboratively optimize resource allocation without centralized data sharing. In this framework, DRL agents in each MEC make local scaling decisions and exchange model parameters with other MECs, rather than sharing raw data. To enhance robustness against malicious server attacks, we employ a committee mechanism that monitors the DFL process and ensures reliable aggregation of local gradients. Extensive simulations were conducted to evaluate the proposed framework, demonstrating its ability to maintain cost-effective resource usage while significantly reducing blocking rates across diverse traffic conditions. Furthermore, the framework demonstrated strong resilience against adversarial MEC nodes, ensuring reliable operation and efficient resource management. These results validate the framework’s effectiveness in adaptive and efficient resource management, particularly in dynamic and varied network scenarios.

## 1. Introduction

With the advent of 5G and the forthcoming 6G networks, there is a vision to create multipurpose and intelligent networks that accommodate both traditional end users and various industry sectors. Advanced virtualization technologies in the mobile core network facilitate network virtualization by changing network elements and functions from hardware-based systems to software-based containers. This transformation offers the necessary flexibility for dynamic resource allocation. Correspondingly, the Third Generation Partnership Project (3GPP), which standardizes mobile communication systems, has outlined the 5G core framework. This framework includes various network functions based on a service-based architecture, where each element is virtualized and runs within virtual machines (VMs) or containers as virtualized network functions (VNF) [1,2].

In this paradigm shift, we have encountered a rising demand for 5G networks, necessitating more efficient management of computational resources within mobile core networks. In other words, instances of network functions must be precisely scaled to accommodate varying customer demands, by either initiating new instances or terminating them based on system load [3]. For example, during peak times with high customer demand, additional instances should be deployed to meet the needs, whereas during off-peak times, instances should be reduced to conserve resources. However, dynamically adjusting computational resources by predicting future demand based on past patterns remains a challenging task.

Numerous research efforts have been directed towards efficiently scaling virtual instances in the 5G core network [4,5,6]. In practice, Kubernetes, a popular container orchestrator in practice, has become a leading cloud manager in recent years, offering Vertical and Horizontal Pod Autoscaling (VPA, HPA) [7,8]. Default VPA in Kubernetes involves adjusting the resources allocated to containers by terminating and redeploying them with the updated resources. Due to this limitation, it has attracted less interest from researchers. In contrast, Default HPA in Kubernetes involves adjusting the number of replicas of the same pod to distribute the load. The default HPA relies on manually configuring threshold values, including CPU utilization, and setting the minimum and maximum number of pods. But these scaling approaches are not adaptable to varying workloads. To address dynamic service demands, various methods described in Section 2 utilizing each base technology have been proposed. Recently, the use of deep reinforcement learning (DRL) has gained significant attention in tackling this problem since DRL is a great tool for decision-making in complex and dynamic environments. Moreover, it can outperform traditional approaches when a (near)-optimal solution is unavailable via a data-driven approach. Hence, such DRL-based approaches can adaptively manage core network resources according to customer demand, addressing both service requirements and resource optimization [9]. Moreover, the integration of the Network Data Analytics Function (NWDAF), which is standardized in 3GPP, and DRL presents a promising avenue for enhancing network optimization and management where the NWDAF, a crucial component of 5G networks, excels in collecting and analyzing data about network performance, state, and user experience [10,11].

To the best of our knowledge, most DRL-based virtual instance scaling in the 5G core network relies on centralized learning. In this approach, state-related information from various network functions in Multi-access Edge Computing (MEC) servers is gathered in a centralized server to train the agent. MEC is a crucial component of 5G and 6G networks, bringing computing capabilities closer to end-users, thereby reducing latency and improving service quality. However, this centralized DRL architecture raises privacy concerns due to the presence of sensitive information. This issue becomes more serious when MEC servers are operated by different service providers, supporting multi-tenants. Conversely, if the DRL agent relies on local MEC server information, there is difficulty in applying trained models to different regions with varying customer demand patterns, resulting in different statistical distributions of input data [12].

To address the aforementioned problem, we suggest using decentralized federated learning (DFL) instead of the traditional DRL-based virtual instance scaling in a centralized manner. DFL enables decentralized training of agents by allowing each agent in MEC servers to train their models locally, without sending data to a central server, thereby preserving data privacy. Therefore, this paper proposes a decentralized federated reinforcement learning (DFRL) framework that leverages the combination of DRL and DFL to address these challenges effectively, aiming for adaptive and efficient virtual instance scaling within MEC environments for 5G core network automation. Moreover, since existing DFL frameworks are vulnerable to Byzantine clients and malicious server attacks [13]—where Byzantine clients may upload incorrect gradients, negatively impacting the global model’s performance and potentially causing training failure—we incorporate a committee mechanism into our proposed DFRL framework. This mechanism addresses Byzantine attacks on MEC servers. A subset of participating MEC servers is appointed as committee members, responsible for monitoring the overall DFL process and ensuring the reliable aggregation of local gradients.

In summary, our proposed framework combines DRL and DFL to address the challenges of resource management in MEC environments while ensuring robust data privacy and protection against Byzantine attacks. Our contributions include developing algorithms for effective DRL training within a DFL paradigm, designing a system architecture to support the DFRL framework with the committee mechanism considering 3GPP standardization, and demonstrating the framework’s efficacy through comprehensive simulations. The detailed contributions of this paper are summarized as follows:

- We explore the effectiveness of NWDAF for gathering states essential for the DRL scheme. Using NWDAF, situated within the 5G core network, can achieve an essential goal in data analysis and long-term management. To design a virtual UPF instances scaling module, we should monitor all the information of the core network such as the number of instances, for better scaling decision [3,14]. AI-based solutions addressing data-centric challenges should be rooted in NWDAF.
- We propose an innovative DFRL framework that combines DRL with DFL to efficiently and adaptively manage UPF instance scaling in MEC environments for 5G core networks. In the proposed framework, DRL agents in each MEC make scaling decisions locally and share model parameters with other MEC servers, rather than sharing their raw data. We utilize the FedAvg [15] algorithm to aggregate their models.
- Despite the strengths of DFL and DRL, inherent limitations exist, particularly concerning data privacy. To tackle potential attacks from malicious MECs and aggressive request distributions, we adopt the incorporation of committee-based mechanisms as a defense strategy. It treats the MEC core network as a node, and in each epoch in the DFL process, some nodes turn into committee members and they are responsible for monitoring the entire DFL training process and ensuring the reliable aggregation of the local gradients.
- We evaluated the proposed framework to determine its effectiveness in maintaining scaling performance under various types of arrival rates. The results indicated that the trained DFRL agent could quickly adjust the number of pods and reduce the blocking rate even when faced with the arrival rates of the evaluation data distribution. Additionally, we conducted experiments by providing the agent with arrival rate data that follow a completely new distribution, different from the traditional Poisson distribution. Moreover, the agent was adjusted to prevent excessive pod scaling in the MEC core network. These findings underscore the adaptability and robustness of the proposed framework in dynamic and varied network conditions.

## 2. Related Work

In the context of 5G and 6G networks, the autoscaling of virtual instances is crucial for efficient resource management. Based on [16], we categorize application scaling theories into non-RL-based theories and RL- and DRL-based theories.

### 2.1. Non-Reinforcement Learning Based Scaling Methods

**Threshold-based Autoscaling**. Threshold-based autoscaling involves setting predefined thresholds for resource utilization metrics such as CPU, memory, and network usage. This method is straightforward to implement and widely used in cloud environments. F. Paraiso et al. [17] presented a component-based Platform as a Service, soCloud, which manages portability, elasticity, provisioning, and high availability. C. Kan [18] proposed DoCloud, which automatically controls Docker container to scale a Web application’s resources. L. Baresi et al. [19] presents a reactive autoscaling policy which consists of a gray-box discrete-time feedback controller to allow containerized applications to scale their resources. However, it has several limitations. First, predefined thresholds may not adapt well to varying workloads, leading to suboptimal scaling decisions. Additionally, to avoid performance degradation, thresholds are often set conservatively, which can result in over-provisioning and increased costs. Furthermore, this method lacks the flexibility to handle complex and dynamic workload patterns effectively.

**Queueing Theory-based Autoscaling**. Queueing theory-based autoscaling applies queueing models to predict resource demands and manage workloads, helping in efficient resource distribution based on expected traffic patterns. Q. Zhang et al. [20] proposed a regression-based approximation of the CPU request and applied it in the queueing network. A. Ali-Eldin et al. [21] concentrated on horizontal elasticity, the power to add or remove resources allocated to a service in the cloud. They proposed two adaptive controllers based on queueing theory, which predict the future request rate of a service. E. B. Lakew et al. [22] focus on their performance models which forecast response latency. Queueing models rely on assumptions about traffic patterns that may not always hold true, leading to inaccuracies. Developing accurate queueing models for complex systems can be challenging and computationally expensive. Additionally, as the system grows, maintaining and updating the queueing models can become increasingly difficult.

**Time Series Analysis-based Autoscaling**. Time series analysis-based autoscaling uses historical data to predict future resource requirements and adjust resources proactively. This method can effectively prevent performance degradation by anticipating demand. P.D. Kaur et al. [23] presented a Quality of Service (QoS)-Aware Resource Elasticity (QRE) framework for dynamically scaling cloud resources to host application components while considering user, application, and system resource heterogeneity and ensuring compliance with user-agreed QoS criteria, with effectiveness demonstrated through experiments on Amazon EC2. H. Fernandez et al. [24] presented an advanced autoscaling system that addresses the limitations of current provisioning systems by leveraging heterogeneous resources and multiple QoS levels, selecting scaling plans based on workload and customer requirements, and demonstrating significant reductions in QoS violations in experiments on public and private infrastructures. E. Kassela et al. [25] propose extending TIRAMOLA, a cloud-enabled framework for the automated resizing of NoSQL clusters, to identify and adapt to different workload types using user-defined policies, enhancing its accuracy in scaling decisions and demonstrating its effectiveness through extensive evaluation on an HBase cluster. However, its effectiveness is highly dependent on the availability of accurate and extensive historical data. Accurate predictions require a large amount of historical data, which may not always be available. Furthermore, time series models may not adapt quickly to sudden and unexpected changes in workload patterns, reducing their reliability in highly dynamic environments. Continuous data collection and analysis also introduce significant computational overhead.

### 2.2. Reinforcement Learning-Based Scaling Methods

Recently, various machine learning techniques, especially RL and DRL, were considered for scaling application resources. RL and DRL methods use machine learning algorithms to learn optimal scaling policies through interaction with the environment, adapting to changing conditions dynamically. These methods have the potential to provide highly adaptive and efficient scaling solutions. J. Rao et al. [26] proposed VCONF, an RL-based approach for automating VM configuration, addressing the challenges of dynamic resource reconfiguration due to changing application demands and resource supply. X. Dutreilh et al. [27] proposed an approach to improve resource dimensioning in cloud computing by integrating RL with techniques for better initialization, convergence speedup, and performance model change detection, and demonstrated its implementation as an automated workflow in a real cloud controller to balance performance and cost-effectiveness. E. Barrett et al. [28] proposed using a temporal difference RL algorithm, specifically Q-learning, within a decision–theoretic framework such as Markov decision processes, to determine the optimal scaling policies for dynamically scaling applications on IaaS clouds, addressing performance interference and resource allocation challenges, and introduce a novel parallel Q-learning approach to mitigate the curse of dimensionality and reduce learning time. L. Schuler et al. [29] propose an RL approach to optimize request-based auto-scaling in serverless computing, demonstrating improved performance over default configurations by learning effective scaling policies for varying workloads. A.A Khaleq et al. [30] introduce an intelligent autoscaling system for microservices in the cloud using an RL technique, which dynamically identifies autoscaling thresholds and enhances response times while maintaining QoS constraints with minimal user intervention. W.F. Villota-Jacome et al. [31] propose SARA and DSARA, RL- and DRL-based mechanisms for intelligent admission control and resource allocation in 5G core network slicing, optimizing resource utilization and service provider profit while meeting the QoS requirements of diverse 5G use cases.

However, they also present considerable challenges. RL and DRL models require extensive training, which can be time-consuming and computationally expensive. These models may take a long time to converge to optimal solutions, especially in complex environments. Moreover, RL and DRL systems can be unstable if not properly tuned, potentially leading to erratic scaling decisions. Nevertheless, traditional centralized DRL approaches require the aggregation of vast amounts of data, including sensitive information from various network functions, raising significant privacy concerns. To address this, we employ DFL over MEC, allowing agents to train collaboratively without compromising privacy. Additionally, to enhance robustness during the DFL process, we implement a committee mechanism.

## 3. Proposed Scaling Method

In this section, we describe our proposed framework that enables MEC in various states to adaptively scale UPF instances using RL. Specifically, adhering to the 3GPP standardization, our framework utilizes the NWDAF to collect data and train the model. Each RL agent within the MEC trains the global RL model using an FL process without a centralized server, which we call decentralized federated reinforcement learning (DFRL). Additionally, to address privacy concerns, particularly regarding potential malicious attacks, the proposed framework incorporates a committee-based defense mechanism.

### 3.1. Overall Architecture

**Figure 1:**
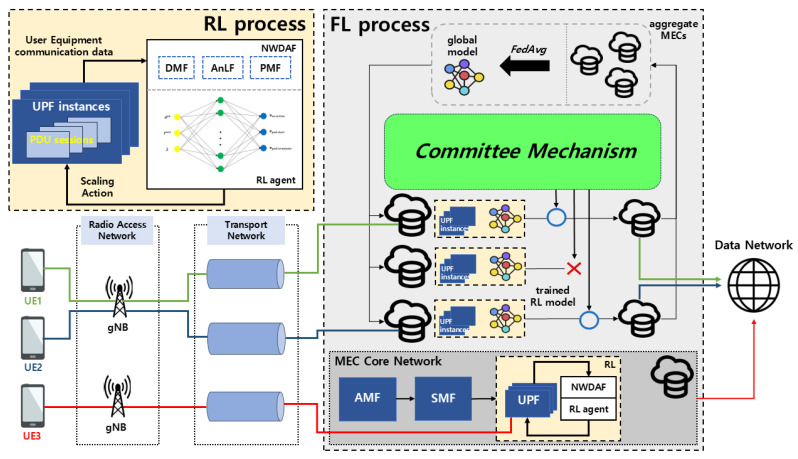
Overall architecture of the proposed framework.

Figure 1 describes the overall scaling method of this architecture where various MECs are deployed to service a 5G network. As following the 3GPP standard of 5G explained in [3], the User Equipment (UE) connects to the Data Network (DN) in a 5G system via a Protocol Data Unit (PDU) session. This process involves the UE first connecting to a gNB in the Radio Access Network (RAN), which then routes the connection through the transport network to the 5G core, eventually reaching the DN. The 5G core comprises several network functions following a service-based architecture. Key functions include the Access and Mobility Management Function (AMF), which handles UE authentication and access control, the Session Management Function (SMF), which manages PDU session states, and the UPF, which is responsible for data forwarding and handling QoS, among other tasks. The control plane is managed by AMF and SMF, while the UPF operates in the user plane, ensuring efficient data routing and processing. In this paper, we call the UPF instance a UPF pod, or simply pod. If there are too few UPF pods, the QoS deteriorates due to insufficient handling capacity for new PDU sessions. Conversely, too many idle UPF pods waste resources and increase operational costs. Thus, the primary objective of scaling UPF pods is to optimize resource usage by adjusting the number of pods based on PDU session demands. Correspondingly, the system aims to balance between maintaining QoS and minimizing resource costs by dynamically scaling the number of UPF pods. Constraints include minimum and maximum limits on the number of UPF pods and the maximum number of sessions a single pod can handle. We assume that UPF pods can initialize immediately, which means the booting time converges to 0.

To efficiently design scaling policies through interaction with the environment and adapt to changing dynamic conditions, we utilize DRL-based autoscaling, as described in [3]. Our architecture, compliant with 3GPP standards, employs the NWDAF to collect UE communication data from UE instances as state information, which are then used to train the RL agent where Data Management Function (DMF), Analytics Function (AnLF), Policy Management Function (PMF) modules in NWDAF are considered. To leverage diverse state information without compromising privacy on MEC servers, we also implement a decentralized federated learning (DFL) process. This allows for the collaborative training of DRL agents across MEC servers without relying on a centralized aggregation server. Additionally, to ensure the reliability of MEC and prevent potential malicious attacks from Byzantine servers, we propose a committee mechanism to select trustworthy MECs.

### 3.2. Decentralized Federated Reinforcement Learning (DFRL) Framework

**Figure 2:**
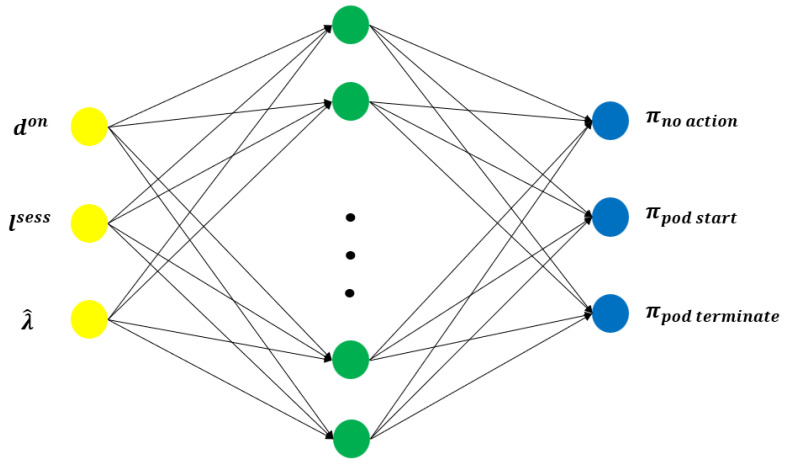
The neural network for the policy π takes the state as input and produces the action probabilities for that specific state. The connections correspond to the weights in θ, and the nodes in the hidden layer apply a non-linear activation function.

Here, we assume that there are *K* MEC servers, denoted as a set $M=\{m_{1},m_{2},\cdots m_{K}\}$ with the core network with multiple UPF instances which connect UE and DN. The list of notations is in List of Notations. For each type of PDU session, we assume that a minimum of $D^{\min}$ pods are initiated, and up to $D^{\max}$ pods can be started. Each pod can manage a maximum of $L^{\mathrm{sess}}$ sessions simultaneously. Let $d_{k,t}^{\mathrm{on}}$ represent the number of active pods in MEC *k* at round *t*. Thus, it holds that $D^{\min}\leq d_{k,t}^{\mathrm{on}}\leq D^{\max}$, and the system can handle up to $D^{\max}L^{\mathrm{sess}}$ sessions. Let $l_{k,t}^{\mathrm{sess}}$ denote the number of activating sessions in the system at time round *t*. Therefore, the system can hold $0\leq l_{k,t}^{\mathrm{sess}}\leq D^{\max}L^{\mathrm{sess}}$ sessions. The following steps summarize the DFRL training process of our proposed system architecture:

1. **Initialization**: Each MEC server initializes their hyperparameters of a DRL agent randomly and resets their environment as in [3]. Then, the system randomly selects the committee MEC set $S_{0}^{c}$ using the proposed committee mechanism, which will be explained later. Other MEC servers become the training MEC set $S_{0}^{b}$. Before implementing a DRL algorithm, it is necessary to represent the issue as a Markov decision process (MDP) problem. This involves defining the state space S, the action space A, and the reward function r:S×A×S→R. Additionally, the complete MDP definition requires the state transition probability p:S×A×S→[0,1] and the discount factor γ∈[0,1]. We use a model-free reinforcement learning method. In the MDP framework, an agent interacts with the environment defined by the MDP. At each decision round *t*, it observes the state $s_{t}\in S$ and, according to its policy $\pi_{t}:S\to A$, takes an action $a_{t}\in A$. As a result, the agent receives a reward $r_{t}$ and observes the next state at the subsequent round. The state at round *t* should include all necessary information for optimal scaling decisions. In our case
  (1) $$s_{k,t}=\left\{d_{k,t}^{\mathrm{on}},l_{k,t}^{\mathrm{sess}},{\hat{\lambda }}_{k,t}\right\}$$
  where ${\hat{\lambda }}_{k,t}$ represents the measured arrival rate of MEC *k* since the previous decision at round *t*. This information is collected by DMF in NWDAF [32,33]. The action space includes three possible actions: start a new pod, terminate an existing pod, or take no action. The agent may start new pods if there is available capacity in the cluster, i.e., $d_{k,t}^{\mathrm{on}}<D^{\max}$, and the agent may only terminate pods if $d_{k,t}^{\mathrm{on}}>D^{\min}$. Termination is considered graceful, meaning the pod waits for all its PDU sessions to close before shutting down and will not accept new sessions during this time. Each agent selects an action through its stochastic policy $\pi_{t}$ in order to maximize their cumulative reward:
  (2) $$a_{k,t}∼\pi_{k,t}\left(a_{k,t}|s_{k,t}\right)\;\mathrm{where}\;a_{k,t}\in A=\left\{‘\mathrm{pod}\;\mathrm{start}’,‘\mathrm{pod}\;\mathrm{terminate}’,‘\mathrm{no}\;\mathrm{action}’\right\}.$$
  The reward function is defined as follows:
  (3) $$r_{k,t}=\left\{\begin{array}{cc} -\kappa {\hat{b}}_{k,t} & \mathrm{if}\;{\hat{b}}_{k,t}>b_{th}\\ -d_{k,t-1}^{\mathrm{on}} & \mathrm{if}\;{\hat{b}}_{k,t}\leq b_{th} \end{array}\right..$$
  where ${\hat{b}}_{k,t}$ is the measured blocking rate since the last decision in MEC *k* at round *t* and $b_{th}$ is the blocking rate threshold as defined by the QoS level, and κ is reward multiplier variable. In the proposed reward structure, ${\hat{b}}_{k,t}$ represents the UE request processing performance, which can be considered as the QoS of the MEC, while $d_{k,t-1}^{\mathrm{on}}$ denotes the number of currently active pods, which is proportional to the operational cost of the MEC. At each round *t*, the goal of each RL agent is to maximize its reward. If ${\hat{b}}_{k,t}$ exceeds the threshold due to an excessive number of requests (the first case of the reward function), the agent receives a significant negative reward proportional to the value of ${\hat{b}}_{k,t}$. This penalty encourages the agent to reduce the blocking rate by providing additional pods, thereby maintaining the desired level of QoS. In contrast, if ${\hat{b}}_{k,t}$ remains below the threshold (the second case of the reward function), the agent receives a significant negative reward based on the value of $d_{k,t-1}^{\mathrm{on}}$. This penalty motivates the agent to minimize the number of unnecessary active pods, thus avoiding resource waste and minimizing the operational cost of the MEC. An LF module in NWDAF [32,33] analyzes UE communication data from the DMF module and refines them as ’state-action-reward’ data for training RL agent. The scalar coefficient κ scales the blocking rate to align it numerically with the $d_{k,t}^{\mathrm{on}}$ value. The logic behind this reward function is to minimize the blocking rate if it exceeds the threshold and minimize the number of pods if it does not. We set κ=15 with many trial-and-error and experiments.
2. **Local RL Agent Training and Update**: In each round, each MEC trains its local RL agent with its UE communication data collected from DMF in NWDAF to maximize the long-term cumulated reward $V^{\pi }\left(s\right)$ with state *s*, defined as
  (4) $$V^{\pi }\left(s\right)=E_{\pi }\left[\sum_{i=0}^{\infty }\gamma^{i}r_{t+i}|s_{t}=s\right].$$
  The optimal policy $\pi^{*}$ is independent of the starting state.
  After the round, each MEC adjusts its scaling strategy with its own RL model by utilizing PMF in NWDAF [32,33]. The PPO algorithm is an actor–critic algorithm [34]. It employs a parameterized policy π(s,θ) as the actor to choose actions, where θ represents the parameter vector. Additionally, the algorithm approximates the value function with V(s,ω), which is parameterized by the ω vector. This value function is utilized to compute the advantage $A^{GAE}$ using the Generalized Advantage Estimation (GAE) method [35]. In PPO, the actor and critic networks are optimized with distinct objectives to achieve stable and efficient learning. Specifically, the actor network is responsible for learning the policy, which determines the actions the agent should take in different states. The purpose of training the actor network is to improve the agent’s decision-making process by gradually adjusting the policy to maximize expected rewards. In this process, PPO uses a clipping mechanism [36] to ensure that policy updates remain within a controlled range, preventing overly large changes that could destabilize learning. On the other hand, the critic network estimates the value function, which predicts the expected cumulative reward for a given state. The critic’s objective is to minimize the prediction error between the estimated value and the actual rewards received, improving the accuracy of the value function over time. By optimizing both the actor and critic together, PPO ensures that the agent not only improves its action selection (through the actor) but also gains a better understanding of the long-term outcomes of its actions (through the critic). This dual optimization enables the agent to learn stable and effective policies while balancing exploration and exploitation in dynamic environments. Although the values in the state are practically constrained by the maximum number of pods $D^{\max}$ and the maximum arrival rate $\lambda^{\max}$, the state space can become so large that it is impractical to store the policy or value function in a computer’s memory. To address this, a neural network with 1 hidden layer consisting of 50 hidden nodes was used to approximate the policy π and the value function *V*, and is summarized in Figure 2 and Table 1. In this context, θ and ω denote the parameter sets of each actor and critic networks, and w denotes a tuple with those two model parameters (θ,ω).
  During each round, each MEC $m_{k}$ trains its local neural model as an RL agent using its UE communication data to minimize the local objective function $F_{k}\left(w_{k}\right)$ with parameter $w_{k}$, which is defined as follows:
  (5) $$F_{k}\left(w_{k}\right)=\frac{1}{N_{k}}\sum_{i=1}^{N_{k}}f\left(w_{k};\xi_{k,i}\right),$$
  where $N_{k}=\left|D_{k}\right|$ are the amount of UE communication data collected at MEC $m_{k}$ and $\xi_{k,i}=\left(s_{k,i},a_{k,i},\pi \left(\cdot |s_{k,i},w_{k,i}\right),r_{k,i},s_{k,t+1}\right)$ are the sample data at time *i* in MEC $m_{k}$ and $f(w_{k};\xi_{k,i})$ is the loss function of basic PPO algorithm [36] which is optimized to minimize the discrepancy between policy improvement and value function prediction using Mean Squared Error (MSE). It should be noted that various loss functions commonly used in federated learning (FL) can be represented as $F_{k}\left(w_{k}\right)$. In this study, the Stochastic Gradient Descent (SGD) method is chosen for the loss function as it is widely adopted in FL, as mentioned in [37]. The time complexity of local training and updates for the PPO-based RL agent are related to the complexity of the policy and value networks [38]. The time complexity of the PPO algorithm increases linearly with the size of the action space and the number of nodes in the hidden layer. In our case, as shown in Table 1, the number of nodes in the network and the size of the action space are small, so the time required for a single update is negligible.
3. **Local Agent Scoring and Selection**: Training agents transmit their local gradients to each committee MEC, which then evaluates these gradients based on a predetermined scoring system. Only those gradients that meet the criteria of the established selection strategy are utilized to construct the global gradient. We will describe the details of the committee mechanism later in Section 3.3.
4. **Aggregation**: The selected MEC becomes an aggregation set $S_{t}^{a}$, and transmits the local model parameters from the agent and updates the global model parameter $\bar{w}$. The widely adopted FedAvg [15] aggregation method is used, formulated as an optimization problem:
  (6) $$\min_{\bar{w}}\left(F\left(\bar{w}\right)=\frac{N_{k}}{N}\sum_{k=1}^{K}F_{k}\left(w_{k}\right)\right),$$
  where *N* is the total number of UE communication data across all MEC servers. The aggregation set broadcasts the updated global model parameters back to m∈M.
5. **Iteration**: This distributed local training and RL agent model parameter aggregation process is repeated until the RL agent thought to be fully trained to adaptive scale pods or iteration round *t* is reached at maximum communication round *T*. Excessive training can lead to overfitting, which significantly degrades the performance of the proposed algorithm. While [3] addressed the volatility of the PPO algorithm by integrating it with a machine learning model, we mitigate this variability through the application of FL. By setting an appropriate value *T*, FL allows us to employ an optimally converged model before the significant performance degradation associated with overfitting occurs, enabling a more efficient training process.

The following algorithm provides a detailed overview of our proposed process, including the DFRL step and committee step.

### 3.3. Committee-Based Architecture

In FL, workers typically do not share their training data with others. Consequently, some workers may be malicious, altering parameters (such as weights or gradients) in their models to undermine the training accuracy of the global model. This type of behavior is commonly referred to as a Byzantine attack [39]. Recent studies reveal that standard FL is highly susceptible to Byzantine attacks by faulty or malicious clients [40]. Even a single attacker can drastically reduce model accuracy from 100% to 0%.

**Figure 3:**
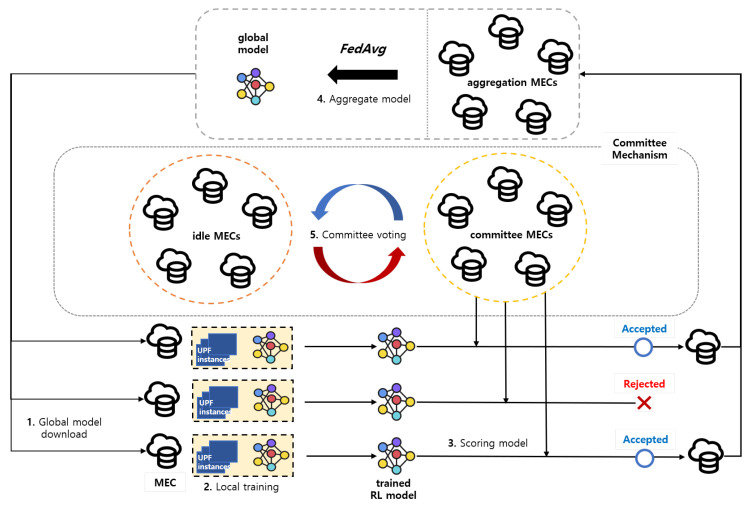
Proposed committee mechanism.

In this section, as in Figure 3, we propose the committee mechanism for the DFRL framework, which is composed of the scoring system, election strategy, selection strategy, and the committee consensus protocol. The committee mechanism strengthens the system’s resilience by blocking malicious MEC from impacting the training process. By ranking MEC servers based on their scores and choosing those around the middle or higher positions, the committee reduces the influence of malicious MEC servers which usually have lower scores [13].

The following steps summarize the committee mechanism process:

- **Global model download**: Each MEC server $m\in S_{t}^{b}$ downloads a global model $\bar{w_{t}}$.
- **Local training**: Each $m\in S_{t}^{b}\cup S_{t}^{c}$ trains its local model to minimize its local objective function with equation 6. After local training, each MEC computes its local gradient ${\hat{g}}_{k,t}$:
  (7) $${\hat{g}}_{k,t}=g_{k}\left(w_{kt},B_{k,t}\right)=\frac{1}{|B_{k,t}|}\sum_{\xi \in B_{k,i}^{t}}\nabla f\left(w_{k,t},\xi \right),$$
  where $B_{k,t}$ is mini-batch for *t* iterations of SGD, which is randomly sampled from $D_{k}$.
- **Scoring model**: The scoring system’s main purpose is to differentiate between honest and malicious gradients by calculating the Euclidean distance. Honest gradients tend to have a lower Euclidean distance compared to the distance between an honest gradient and a malicious one. Consequently, MEC servers uploading honest gradients receive higher scores, while those uploading malicious gradients receive lower scores. Assume the local gradient on the *k*-th training MEC at round *t* is denoted as ${\hat{g}}_{k,t}=g_{k}\left(w_{k,t};B_{k,t}\right)$, and the local gradient on the *c*-th committee MEC at round *t* is ${\hat{g}}_{c,t}=g_{c}\left(w_{c,t};B_{c,t}\right)$. The score $P_{k}^{c}$ of the *k*-th training MEC, assigned by the *c*-th committee MEC, is calculated as follows:
  (8) $$p_{k}^{c}=\frac{1}{∥{\hat{g}}_{k,t}-{\hat{g}}_{c,t}{∥}_{2}^{2}}.$$
  The final score of the *k*-th training MEC is defined as follows:
  (9) $$P_{k}=\frac{1}{\frac{1}{\left|S_{c}\right|}\sum_{c\in S_{c}^{t}}{∥{\hat{g}}_{k,t}-{\hat{g}}_{c,t}∥}_{2}^{2}}=\left|S_{c}\right|\sum_{c\in S_{c}}\frac{1}{p_{k}^{c}}.$$
  The scoring principle is based on the Euclidean distance between local gradients ${\hat{g}}_{,t}$ and ${\hat{g}}_{c,t}$. Typically, Byzantine attackers replace some local gradients with malicious ones, which increases the Euclidean distance between these and the honest gradients. Thus, when the proportion of malicious MEC is tolerable, their scores will be lower than those of the honest MEC. In non-attack scenarios, the score indicates the degree of MEC heterogeneity, with a higher score representing higher heterogeneity.
- **Aggregate model**: Committee MEC servers select local gradients with higher scores to construct the global gradient. Gradients similar to the committee gradients in Euclidean space participate in constructing the global gradient. We sort the local gradients by their scores and accept the top α% of them, focusing on robustness. This approach ensures a more robust aggregation process by choosing gradients close to those of the committee, assuming the committee is honest. However, this makes it hard for MEC servers with significant differences to be selected, potentially lowering performance.
- **Committee voting**: This ensures the honesty of committee set $S_{t}^{c}$. Committee members reach a consensus on their decisions, which relies on the majority. Malicious MEC servers in the committee can interfere with decision-making, so it is crucial to have more honest than malicious members. Malicious MEC servers generally have low scores, so to prevent malicious members from being included as committee members, the committee members should be selected primarily from those with high scores. However, if the committee is composed solely of the highest-scoring members, the global model’s learning direction would be overly influenced by the initial committee members. This aligns with the intuition that committee members should represent the majority. Thus, we sort training MEC servers by their scores and select those in the middle β% score position as new committee MEC servers.

The following pseudocode represents a committee-based algorithm.

The overall time complexity of our framework (Algorithm 1) is *O* (DFRL process) + *O* (Algorithm 2). As previously noted, the complexity of the RL agent is negligible due to the model size in the proposed scenario. Therefore, the time complexity of the DFRL process is determined by *T* and *K*, while the complexity of Algorithm 2 is determined solely by *K*. Thus, the time complexity of the entire framework can be expressed as $O(TK+K^{2})$.
**Algorithm 1** DFRL training loop1:**for** $m_{1,2,\cdots ,k}\in M$ **do**2:      initialize local RL agent and environment.3:**end for**4:**for** t=0 to *T* **do**5:      **for** $m_{1,2,\cdots ,k}\in M$ **do**6:          download global model parameter ${\bar{w}}_{t-1}$.7:          Get action from agent: $a_{k,t}\leftarrow$ Sample $\pi_{k,t}\left(s_{k,t},\bar{w_{t-1}}\right)$.8:          Execute action $a_{k,t}$ to scale the cluster in MEC $m_{k}$.9:          Observe the new state $s_{k,t+1}$ and performance measures during DFL round.10:        Compute reward $r_{k,t}$ from the measurements using Equation (3).11:        agent update local RL agent using PPO update Algorithm with Equation (6).12:    **end for**13:    get new aggregation set $S_{t}^{a}$ with Algorithm 2.14:    aggregate all $w_{t}$ in aggregation set $S_{t}^{a}$ and generate new global model ${\bar{w}}_{t}$ using FedAvg [15].15:    t←t+116:**end for**

**Algorithm 2** Committee mechanism
  **Input:** *t*, $w_{t}$ from all MECs m∈M
  **Output:** new aggregation MEC set $S_{t+1}^{a}$, new committee MEC set $S_{t+1}^{c}$
1:**for** $c\in S_{t}^{c}$ **do**2:      **for** $k\in S_{t}^{b}$ **do**3:            Committee *c* broadcasts score $p_{k}^{c}$ to other committee MECs.4:      **end for**5:
**end for**
6:**for** $c\in S_{t}^{c}$ **do**7:      **for** $k\in S_{t}^{b}$ **do**8:            Committee *c* calculates the total score $P_{k}$ of MEC *k*.9:      **end for**10:
**end for**
11:Choose MEC servers $a\in S_{t+1}^{a}$ with the top α% of their score among $S_{t}^{b}$.12:Choose MEC servers $c\in S_{t+1}^{c}$ with the middle β% of their score among $S_{t}^{b}$.13:Return $S_{t+1}^{a}$.


## 4. Evaluation

In this section, we evaluated the effectiveness of the proposed framework through simulation-based experiments. First, we implemented the benchmark of the proposed framework and conducted evaluations by comparing the scaling performance according to the varying arrival rate. Then, we conducted experimental results on the defense performance against Byzantine attacks from malicious servers.

### 4.1. Environment Setting

We slightly adjust the simulator in [3], which has a multi-distributed MEC servers serving 5G network using Python 3.9.7. In our experiments, the simulation program was developed using Python. To implement the RL agents, we utilized PyTorch 2.1.1, which allows us to construct neural network model and train the RL agents within the system. The RL environment was managed using Gymnasium 0.29.1, a robust framework for RL environments that provided the necessary tools to simulate dynamic network conditions and interactions between the agents and the environment. For more detailed hyperparameters of our simulator are listed in Table 2. There are *K* MEC servers and each MEC server has a core network with multiple nodes that have PDU sessions which make data transmission occur between UE and DN. When UE arrives, a PDU session is initiated if the system has available capacity; otherwise, the request is blocked. This simulator is equipped to simulate the each arrival of UE as a Poisson process with a specific rate at a given time, but the exact arrival of UE can not be known in practical scenarios. Here, we assume that the arrival rate of UE makes a periodic function:

(10) $$\lambda_{k,t}^{\mathrm{train}}=A_{k}+B_{k}\sin(\frac{1}{C_{k}})\pi t,$$

with random value $A_{k},B_{k},C_{k}$ that $0\leq B_{k}\leq A_{k}\leq 250$ represent the amplitude and the mean of arrival rate, $1\leq C_{k}\leq 10$ represents the period. More detailed parameters of our simulation are shown in Table 2.

### 4.2. Convergence Analysis

In this subsection, we evaluate the convergence of our proposed DFRL framework. We designed our multi-MEC simulator illustrated in Section 4.1. All MEC servers have one DRL agent and train their agent via Algorithm 1. At each round, we collect rewards from all the DRL agents and average them to show the convergence.

**Figure 4:**
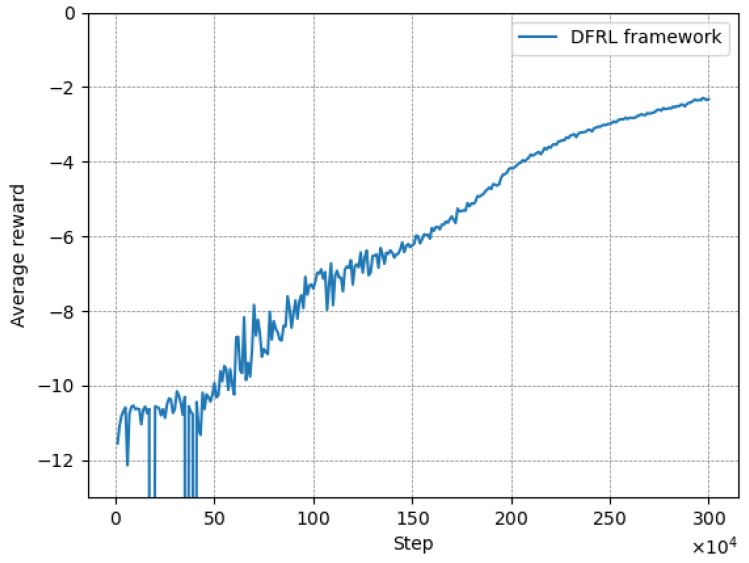
Average reward with proposed DFRL framework.

In Figure 4, at the early stage of evaluation, the average rewards of our framework fluctuate significantly because the agents are still in the exploration phase, and there are insufficient data in the experience replay buffers for effective training. In this phase, the DRL agent can not provide the scaling action properly, leading to too many or too less pod scales, resulting in a poor reward. As the number of training episodes increases, the agents’ average rewards begin to gradually improve. Ultimately, our algorithm reached a stable positive reward and the fluctuation was significantly down. Thus, we conclude that our trained agents can provide scaling action properly while maintaining a request blocking rate under threshold value, with no wasted core network resources.

**Figure 5:**
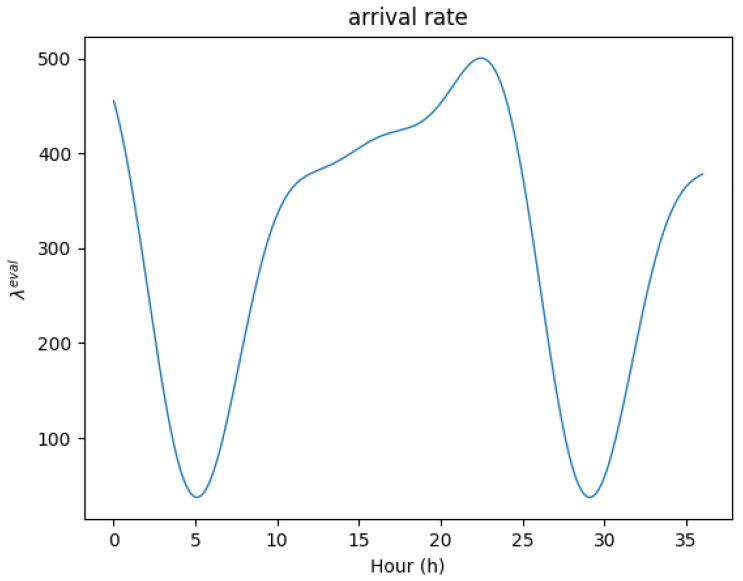
The value of $\lambda_{t}^{\mathrm{eval}}$.

To evaluate our framework, we adapt an equation from [3] which presents mobile user traffic [41] as the evaluation arrival rate:

(11) $$\begin{array}{cc} \lambda_{t}^{\mathrm{eval}} & =330.07620+171.10476\sin\left(\frac{1}{12}\pi t+3.08\right)\\ \; & +\;100.19048\sin\left(\frac{1}{6}\pi t+2.08\right)\\ \; & +\;31.77143\sin\left(\frac{1}{4}\pi t+1.14\right) \end{array}$$

and the value of $\lambda_{t}^{\mathrm{eval}}$ is represented in Figure 5.

**Figure 6:**
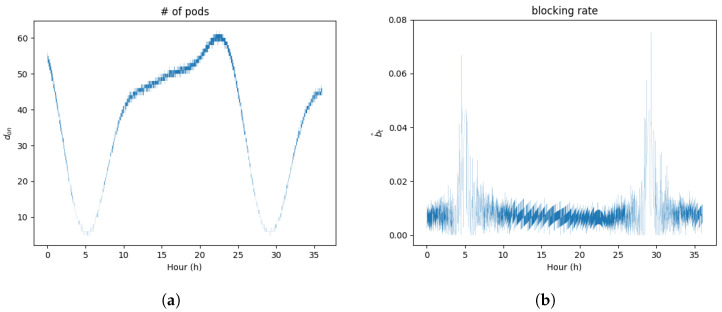
Scaling performance of proposed DFRL framework. (**a**) Pod count $d_{\mathrm{on}}$. (**b**) Blocking rate $\hat{b}$.

The goal of the RL agent in the proposed framework is to minimize cost while maintaining the system’s QoS. As mentioned before, the reward function simultaneously considers the number of active pods and the blocking rate, and in an MEC system, we remind that keeping the request-blocking rate low is synonymous with maintaining the system’s QoS, and minimizing the number of activated pods corresponds to minimizing the system’s cost. Figure 6 illustrates the real-world performance of the proposed framework in MEC environments. Figure 6a shows the scaling of pods in response to request $\lambda^{\mathrm{eval}}$. Compared with Figure 5, we can observe that the proposed framework adjusts the number of pods according to the incoming requests. Figure 6b depicts the blocking rate of requests. With the exception of hour 5 and hour 30, when the number of requests was too low to determine precise scaling actions, the blocking rate is maintained below the blocking rate threshold $b_{\mathrm{th}}$ for nearly all times. Therefore, it is evident that the proposed framework faithfully achieves the goal of minimizing cost while maintaining QoS in the MEC environment.

### 4.3. Different Distribution of Arrival Rate

**Figure 7:**
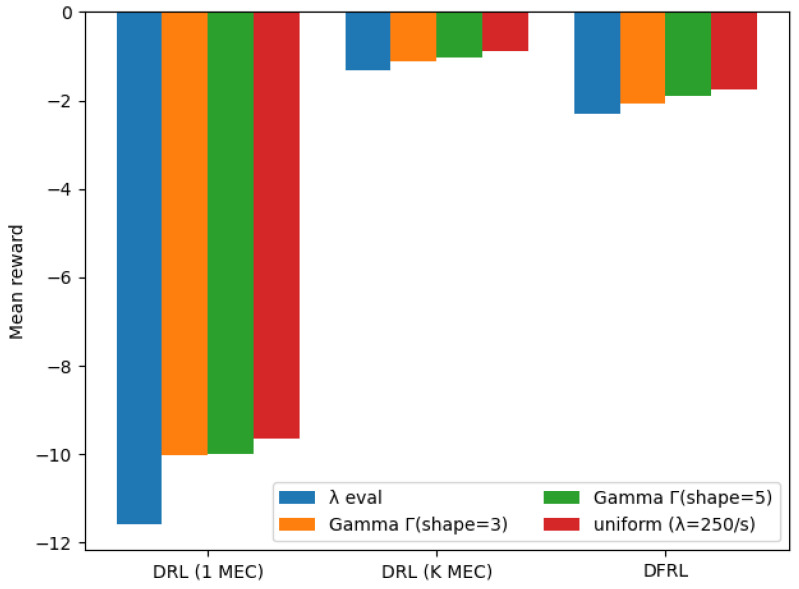
Mean reward results using a different distribution of arrival rate.

In the previous section, we assume that the arrival rate of UE follows a periodic function. However, in reality, the assumption that the arrival rate of UE follows a periodic function may not always hold [42]. To explore the impact of different distributions on the accuracy of our DFRL framework, we perform a sensitivity analysis using simulations with different distributions of arrival rates. We use alternative distributions, which are gamma distribution with shape parameter 3 and 5, and uniform distribution. To evaluate the performance in comparison, we assess the results of the traditional DRL method without the application of the FL technique. In our setup, the DRL agent operates in a single MEC, and we create two different benchmarks based on the amount of data used for training: (1) data collected from only one MEC (i.e., local training) and (2) data aggregated from all *K* MECs (i.e., centralized training). The simulated average rewards for different distributions of arrival rates are shown in Table 3 and depicted in Figure 7.

For the DRL agent trained using data from only a single MEC, the average reward is observed to be very low. This indicates that the agent is unable to appropriately select scaling actions based on the measured arrival rates, leading to inadequate adjustments in the number of pods. As a result, the system either experiences a high blocking rate due to an insufficient number of pods or faces overprovisioning when there are too many pods. The agent remains in the exploration phase of DRL, demonstrating insufficient training due to the lack of enough data, and ultimately shows poor scaling performance. In contrast, the DRL agent trained with the aggregated training data from all MECs (i.e., centralized training) achieved the highest average reward. This suggests that the agent learned from a much wider range of scenarios compared to when it was trained with data from a single MEC, gaining the ability to respond adaptively and flexibly to diverse situations. However, since this approach requires aggregating data from all MEC servers in one location for training, it can potentially cause data privacy concerns and create networking and computing bottlenecks at the centralized server. Therefore, while this benchmark may provide an upper bound on performance, it is not scalable or practical for real-world applications. In comparison, our framework demonstrated scaling performance similar to that of the DRL agent trained with aggregated data from all MEC servers across various arrival rate distributions. By utilizing distributed learning, our framework avoids the need to centralize data, thereby mitigating the previously mentioned data privacy issues and computing/networking bottlenecks while still achieving appropriate scaling actions. These results suggest that our framework is a more practical and effective choice than the traditional DRL methods in a multi-MEC scenario.

### 4.4. Simulation with Malicious MEC Servers

In this subsection, we test the robustness of our committee-based algorithm for Byzantine attacks from malicious MEC servers compared with the DFRL framework with no Byzantine defenses. In our simulator, to imitate the back-gradient attack, malicious MEC servers share their local agent parameter adding significant Gaussian noise which has the purpose of reducing the global model performance. We consider three hyperparameters in committee mechanism, α, β, ϵ is a portion of each aggregator MEC servers, committee MEC servers, and malicious MEC servers, respectively. We divide this evaluation into three sub-tests, and for each sub-test, we set two hyperparameters with a fixed value and vary the other hyperparameter as follows:

1. varying committee ratio β={10,20,30,40,50}, fixed α=40, ϵ=10.
2. varying aggregator ratio α={10,20,30,40,50}, fixed β=40, ϵ=10.
3. varying malicious ratio ϵ={10,20,30,40,50}, fixed α=40, β=40.

**Figure 8:**
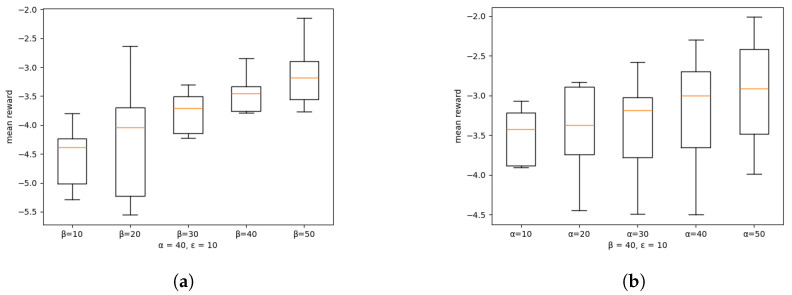
Boxplot showing the distribution of mean rewards across different committee and aggregator ratio. The boxes represent the interquartile range (IQR), with the median indicated by the horizontal line inside each box. Whiskers extend to 1.5 times the IQR. (**a**) Different committee ratio. (**b**) Different aggregator ratio.

**Figure 9:**
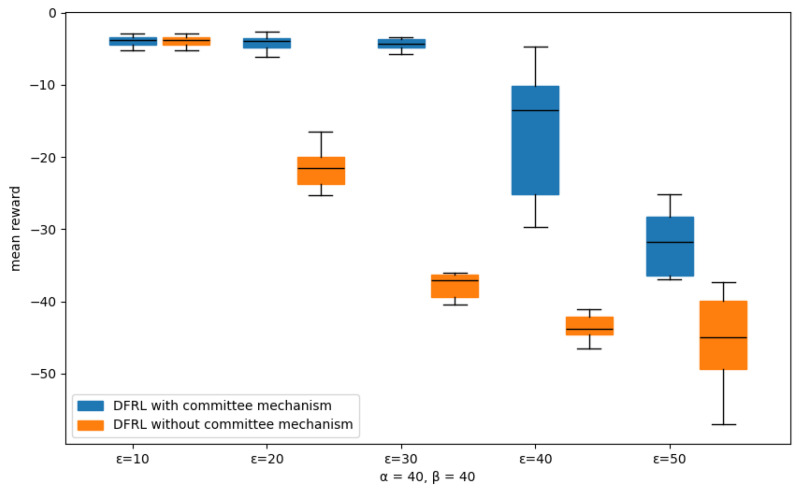
Boxplot showing the distribution of mean rewards across different malicious MEC ratios compared with committee-based and non-committee-based DFRL frameworks.

In sub-tests, we average the rewards from all MEC servers as scaling performance with our committee-based DFRL framework. Also, we run sub-test 3 with a DFRL framework with no committee mechanism and observed the results of the non-committee framework. We run each sub-test 10 times and average those results, which are illustrated in Figure 8 and Figure 9.

Figure 8a demonstrates that increasing the number of committee members by enough can positively impact global performance. Expanding the number of committee members can enhance the robustness of the committee and prevent the training and aggregation stages from being dominated by a small number of members.

Figure 8b shows that appropriately increasing the number of aggregator members can also positively impact global performance. The condition for being selected as an aggregator member is to receive high scores from committee members. In a typical flow where a normal committee is formed, honest members are more likely to receive higher scores from the committee members compared to malicious members, increasing their chances of being selected as aggregator members. By increasing the number of aggregator members, more honest members are likely to be chosen, thereby enhancing the robustness of the global performance.

Figure 9 shows that if the number of malicious members is high, global performance significantly deteriorates in most situations. Increasing the number of committee members raises the likelihood of malicious members being included in the committee. This results in honest members receiving lower scores from the committee, preventing them from being selected as aggregator members. The probability of forming an aggregator composed of malicious members increases, leading to a significant drop in global performance. Similarly, increasing the number of aggregator members can also raise the likelihood of malicious members being included among them, potentially causing further degradation in global performance. Finally, the result shows that the proposed committee mechanism is more robust than a non-committee framework with every ϵ value. The non-committee-based frameworks can not protect their global performance when the malicious ratio ϵ meets only 20%. However, our DFRL framework with a committee-based mechanism can maintain their global performance even ϵ is more than 30%. In the experimental results, it can be observed that the global performance significantly decreases when the proportion of malicious entities exceeds 40%. This decline is attributed to the system being entirely compromised by the attack, preventing it from taking appropriate scaling actions.

## 5. Conclusions

In this paper, we presented a DFRL framework enhanced with a committee mechanism, aiming for robust and efficient virtual instance scaling in MEC environments for 5G core network automation. Our experimental results showed that the proposed framework outperforms traditional approaches by maintaining high network performance and achieving resilience under various operational conditions, including different traffic patterns and the presence of adversarial MECs. Qualitatively, the committee mechanism offers a significant advantage: it proactively identifies and excludes malicious MEC participants, thereby enhancing both security and model stability. This self-regulating feature sets our framework apart from others that either lack strong adversary detection capabilities or struggle to maintain performance consistency in the presence of security threats. By minimizing the likelihood of harmful decisions in the DRL-based decision-making process, our framework introduces an additional layer of control over actions driven by probabilistic policies, though the complete elimination of undesirable actions remains a challenge. Furthermore, the framework demonstrates scalability across a variety of network configurations and operational environments, emphasizing its versatility and potential applicability in future 5G and beyond network deployments. While our results already showcase clear performance improvements, future research should focus on refining the probabilistic aspects of the DRL component to further reduce risks associated with adversarial attacks and stochastic behaviors. This ongoing evolution holds the potential to provide even greater robustness and security for MEC-based automation.

## Figures and Tables

**Table 1:** DRL hyperparameters.

neural network hidden layers	1
node count in each hidden layer	50
learning rate	0.0001
epochs	5
batch size	32
PPO clipping parameter	0.1
GAE parameter	0.9
activation function	ReLU

**Table 2:** Environment hyperparameter setting.

number of MECs (K=|M|)	50
minimum number of Pods in MEC ($D^{\min}$)	2
maximum number of Pods in MEC ($D^{\max}$)	100
PDU session service rate	1/s
requests blocking rate threshold ($b_{th}$)	0.01
scaling decision time interval (Δt)	1s

**Table 3:** Mean reward results using a different distribution of arrival rate.

Distribution	DRL (1 MEC)	DRL (*K* MECs)	DFRL (*K* MECs)
$\lambda_{t}^{\mathrm{eval}}$ (Equation (11))	−11.58974	−1.32819	−2.30002
gamma distribution (Γ with shape 3)	−10.02404	−1.12015	−2.07609
gamma distribution (Γ with shape 5)	−9.99494	−1.02000	−1.90180
uniform distribution (λ=250/s)	−9.65637	−0.88085	−1.75417

## Data Availability

Data are contained within the article.

## List of Notations

**Notations for the environment**	
*K*	number of MEC servers
$D^{\min}$	minimum number of pods
$L^{\mathrm{sess}}$	maximum number of PDU sessions per pod
*t*	current episode round
M	Whole MEC servers set
$D^{\max}$	maximum number of pods
$b_{th}$	blocking rate threshold
*T*	maximum number of episode round
**Notations for the observation**	
$d_{k,t}^{\mathrm{on}}$	number of running pods in MEC *k* at round *t*
${\hat{\lambda }}_{k,t}$	measured arrival rate in MEC *k* at round *t*
$l_{k,t}^{\mathrm{sess}}$	number of running sessions in MEC *k* at round *t*
${\hat{b}}_{k,t}$	measured blocking rate in MEC *k* at round *t*
**Notations for the MDP**	
S	state space set of MDP
A	action space set of MDP
$\pi_{k,t}$, $\pi^{*}$	policy function in MEC *k* at round *t*, optimal policy
p	transition probability
κ	reward multiplier
$s_{k,t}$	state in MEC *k* at round *t*
$a_{k,t}$	action in MEC *k* at round *t*
$r_{k,t}$	reward in MEC *k* at round *t*
γ	discounting factor
*V*	value function
**Notations for the committee mechanism**	
$S_{t}^{a}$	aggregation MEC set
$S_{t}^{c}$	committee MEC set
$w_{k,t}$	model parameters in MEC *k* at round *t*
f(w;ξ)	Overall loss function of RL
${\hat{g}}_{k,t}$	local gradient in MEC *k* at round *t*
$p_{k}^{c}$	score of MEC *k* calculated from committee *c*
$S_{t}^{b}$	training MEC set
θ, ω	parameter vectors of policy and value function
$\bar{w}$	global model parameter
F(w)	Local objective function
$B_{k}$	randomly sampled mini-batch from $D_{k}$
$P_{k}$	total score of MEC *k*
